# Colonization of Non-biodegradable and Biodegradable Plastics by Marine Microorganisms

**DOI:** 10.3389/fmicb.2018.01571

**Published:** 2018-07-18

**Authors:** Claire Dussud, Cindy Hudec, Matthieu George, Pascale Fabre, Perry Higgs, Stéphane Bruzaud, Anne-Marie Delort, Boris Eyheraguibel, Anne-Leïla Meistertzheim, Justine Jacquin, Jingguang Cheng, Nolwenn Callac, Charlène Odobel, Sophie Rabouille, Jean-François Ghiglione

**Affiliations:** ^1^CNRS, UPMC Univ Paris 06, UMR7621, Laboratoire d’Océanographie Microbienne (LOMIC), Observatoire Océanologique de Banyuls, Sorbonne Université, Banyuls-sur-Mer, France; ^2^CNRS/UM, UMR5221, Laboratoire Charles Coulomb (L2C), Montpellier, France; ^3^Symphony Environmental Ltd., Hertfordshire, United Kingdom; ^4^Université de Bretagne-Sud, Institut de Recherche Dupuy de Lôme (IRDL), UMR CNRS 6027, Lorient Cedex, France; ^5^CNRS, UMR6296, SIGMA Clermont, Institut de Chimie de Clermont-Ferrand (ICCF), Université Clermont Auvergne, Clermont-Ferrand, France; ^6^CNRS, UPMC Univ Paris 06, UMR7093, Laboratoire d’Océanographie de Villefranche (LOV), Sorbonne Universités, Villefranche-sur-Mer, France

**Keywords:** plastic pollution, biofouling, microbial ecotoxicology, plastisphere, biodegradable plastics

## Abstract

Plastics are ubiquitous in the oceans and constitute suitable matrices for bacterial attachment and growth. Understanding biofouling mechanisms is a key issue to assessing the ecological impacts and fate of plastics in marine environment. In this study, we investigated the different steps of plastic colonization of polyolefin-based plastics, on the first one hand, including conventional low-density polyethylene (PE), additivated PE with pro-oxidant (OXO), and artificially aged OXO (AA-OXO); and of a polyester, poly(3-hydroxybutyrate-co-3-hydroxyvalerate) (PHBV), on the other hand. We combined measurements of physical surface properties of polymers (hydrophobicity and roughness) with microbiological characterization of the biofilm (cell counts, taxonomic composition, and heterotrophic activity) using a wide range of techniques, with some of them used for the first time on plastics. Our experimental setup using aquariums with natural circulating seawater during 6 weeks allowed us to characterize the successive phases of primo-colonization, growing, and maturation of the biofilms. We highlighted different trends between polymer types with distinct surface properties and composition, the biodegradable AA-OXO and PHBV presenting higher colonization by active and specific bacteria compared to non-biodegradable polymers (PE and OXO). Succession of bacterial population occurred during the three colonization phases, with hydrocarbonoclastic bacteria being highly abundant on all plastic types. This study brings original data that provide new insights on the colonization of non-biodegradable and biodegradable polymers by marine microorganisms.

## Introduction

Within a few decades, plastic has become the biggest form of pollution in the world’s oceans (80% of marine litter consists of plastic) due to its very slow degradability and the growing accumulation of human waste products (Gewert et al., 2015). When released into the environment, plastic litter is fragmented by both physical and chemical processes into small pieces (<5 mm), commonly referred to as “microplastics" (MPs) (Barnes et al., 2009). MPs represent more than 90% of the total counts of plastic debris at the sea surface (Eriksen et al., 2014).

At sea, plastics are almost immediately coated by inorganic and organic matter (so called the “conditioning film”), which is then rapidly colonized by microorganisms that form a biofilm on their surfaces (Loeb and Neihof, 1975; Cooksey and Wigglesworth-Cooksey, 1995). Bacterial biofilms are defined as surface-associated bacterial communities which are embedded within an exopolymeric substance matrix (EPS) (Costerton et al., 1995). These natural assemblages act as a form of protection, nutritive resource, offer metabolic cooperativity, and an increase in the possibility of gene transfer among cells (Davey and O’toole, 2000). The successive phases of biofilm formations are well described within marine waters on artificial (glass, acryl, and steel) or natural surfaces (rocks and algae) (Dang and Lovell, 2000; Salta et al., 2013). First, the “primo-colonization” describes the occupation of the surface by pioneer bacteria through reversible attachment, where they interact with the conditioning film and form the first layer of the initial biofilm. Second, the “growth phase” promotes irreversible attachment by active mechanisms such as the formation of pili, adhesion proteins and EPS produced by secondary species, which induce modifications in the properties of the substratum. Third, the “maturation phase” occurs through diverse, competitive or synergistic interactions between cells, with either further recruitment or loss of species (Lorite et al., 2011).

Very few studies have so far described the formation of biofilms on plastics in marine environments. Early stage processes were followed on polyethylene (PE)-based plastic bags or MPs during 3 weeks in seawater (Lobelle and Cunliffe, 2011) and in sediments (Harrison et al., 2014). Two studies are available on longer-term biofilm formation on the surface of PE or PE terephthalate (PET) in marine environment, which were carried out over a 6-month period (Webb et al., 2009; De Tender et al., 2017). Only one study has so far compared biofilm formation on PE with that observed on so-called “biodegradable” plastics made of starch-based biopolymer-PET blend (Mater-Bi N°014), conducted during 1 month in marine environment (Eich et al., 2015). These studies were mostly based on scanning electron microscopy (SEM) observations and taxonomic identification, but none of them focused on bacterial abundance and activity, meaning that populations and community dynamics in these biofilms remains largely unknown. Moreover, the formation of a biofilm was depicted as strongly dependent on substrate properties including hydrophobicity/hydrophilicity, structure, and roughness (Lorite et al., 2011), which were never taken into account in studies exploring marine environment.

Polyethylene dominates the composition of plastic waste at sea surface, followed by polypropylene (PP) and polystyrene (PS) (Auta et al., 2017). The stable aliphatic chains in PE make it a very recalcitrant material (Tokiwa et al., 2009). Within the frame of sustainable development, a wide range of potentially biodegradable plastics were developed and classified into two major groups depending on the mode of biodegradation pathway: “OXO-biodegradable” and “hydro-biodegradable” (Vázquez-Morillas et al., 2016). The former are polyolefin-based polymers (generally PE) with pro-oxidant additives (OXO; for OXO-degradable polymer). In case of release in the environment, the additive accelerates abiotic oxidation process by heat and/or UV light, a phenomenon that can be simulated by artificial aging of the OXO (AA-OXO, for artificially aged OXO). If the initial formulation of OXO is recalcitrant to biodegradation, the oxidized AA-OXO can be further biodegraded by oxidative mechanisms (Koutny et al., 2006; Eyheraguibel et al., 2017). Several studies on OXO pre-oxidized films showed 50 to 80% mineralization under half to one and a half year of incubation (Jakubowicz, 2003; Chiellini et al., 2007) or between 12 and 24% mineralization after 90 days of incubation (Ojeda et al., 2009 and Yashchuk et al., 2012). Hydro-biodegradable plastics are based on polymers that can be biodegraded by hydrolytic mechanisms (Nampoothiri et al., 2010). They include cellulose, starch and more generally polyesters, such as polyhydroxyalkanoates (PHA). Because PHA are polyesters made by bacteria for intracellular storage of carbon and energy, they received considerable attention as promising biodegradable polymers to substitute for traditional plastics, with mechanical properties similar to various synthetic thermoplastics (Corre et al., 2012; Elain et al., 2015, 2016). Various bacteria were shown to degrade AA-OXO or PHA in different conditions (Tokiwa and Calabia, 2004; Sudhakar et al., 2008; Ammala et al., 2011).

In this study, we characterized the biofilm colonization phases on PE, OXO-degradable polymer with (AA-OXO) or without (OXO) artificial-aging, and poly(3-hydroxybutyrate-co-3-hydroxyvalerate) (PHBV) as PHA representative. Each polymer type was separately incubated and its evolution monitored during 6 weeks in natural seawater from Banyuls Bay (NW Mediterranean Sea). The dynamics of bacterial biofilms was described in terms of changes in abundance, diversity and heterotrophic activity, together with changes in polymer surface physical properties (contact angle and roughness).

## Materials and Methods

### Polymer Samples Preparation and Design of the Incubation Experiments

In this study, we used four types of polymer: PE corresponded to commercially available commodity film grade low-density PE resin Borealis FA6224, which had the following characteristics: density = 0.922 g cm−^1^, average molecular weight M_W_≈97,000 kg mol^−1^, with a melt-flow index (MFI) = 2.1 g/10 min (190°C, 2.16 kg). OXO was made of the same PE formulation but additivated with D_2_W OXO based on manganese and iron (provided by Symphony Environmental Ltd., United Kingdom). AA-OXO was made of same OXO formulation but thermally aged for 180 days in an aerated oven at 70°C, which resulted in fragmentation, loss in mechanical properties and increase in oxidation level as depicted by absorbance increase at 1,712 cm^−1^ determined by micro-FTIR spectroscopy reaching more than x/100 (where x was the film thickness). The level of x/100 was previously demonstrated as a prerequisite for biodegradability of OXO, as already demonstrated for *Rhodococcus rhodochrous* and described in the French Agreement Association Française de Normalisation (AFNOR) (2012) PE, OXO, and AA-OXO were extruded at 180°C using a laboratory scale Rondol linear 18 mm blown film line.

Poly(3-hydroxybutyrate-co-3-hydroxyvalerate) (provided by University of South Brittany, France) had the following characteristics: density = 1.25 g cm^−1^, average molecular weight M_w_≈400 kg mol^−1^, with a MFI = 3.6 g/10 min (210°C, 2.16 kg). This grade has been comprehensively characterized in a previous paper (Corre et al., 2012). Prior to compression molding, the PHBV pellets were dried over 12 h under vacuum at 60°C to minimize the hydrolytic PHBV degradation during processing and compression molded in a Carver^®^ hydraulic press at 180°C under a pressure of 10 metric tons for 3 min.

The thickness of polymer films was 200 μm for PHBV and 100 μm for PE, OXO and AA-OXO. Each sample was a circular piece of 9 mm diameter (area = 63.6 mm^2^), except for AA-OXO that was constituted of irregular fragments of mean area of 13.9 ± 4.8 mm^2^ after artificial aging. Each polymer sample was cleaned with 70% ethanol and washed with sterile seawater (SSW) before incubation.

We used five identical aquariums consisting in trays with a 1.8 L capacity (Sodispan, Spain), in which 1.5 L seawater was continually renewed by direct pumping at 4 m depth in Banyuls bay, close to the SOLA observatory station (NW Mediterranean Sea, France). A flow rate of 50 mL min^−1^ was chosen to ensure a sufficient renewal of natural bacteria (every 30 min) and an homogeneous distribution of the plastic pieces in the aquariums during the entire experiment. Each aquarium contained polymer pieces of one of the composition (PE, OXO, AA-OXO, and PHBV), except one aquarium used as control, containing only circulating seawater (hereafter called “control aquarium”). Pieces of each polymer type were put in the 18th of January, 2016 and sampled after 7, 15, 22, 30, and 45 days. Aquariums were kept in the dark to avoid UV-driven degradation of the polymers. Throughout the experiment, seawater temperature (between 12.5 and 13.5°C) and salinity (38.5) in the aquariums were similar to seawater from Banyuls bay.

### Atomic Force Microscopy

Atomic force microscopy (AFM) was performed on each sample to get resolved picture of the colonization and accurate insight of the surface state of the polymer. At each sampling time, one piece of each polymer was rinsed with SSW and fixed for at least 1 h at 4°C with 1% (v/v) glutaraldehyde (final concentration) before freezing. At least three 40 × 40 μm^2^ areas images were acquired for each sample using a Nanoscope V (Bruker instruments, Madisson, WI, United States) in dynamic mode (Binnig et al., 1986) and standard silicon probes (Bruker, TESP-V2). Root mean square (RMS) roughness of the polymer surface were measured on height images of 40 × 40 μm^2^ with Gwyddion software, using masks to remove remaining bacterial cells and other organic deposits from the measurements. Boxes of gradual sizes (10, 20, 30, and 40 μm) were used to estimate RMS standard deviation and to check the dependence of the RMS on the lateral size of the picture. On every sample, a plateau was reached at 30 μm, which validates the use of RMS measured on 40 μm size to characterize the surface state of the sample.

Since surface state characterization is likely to be affected by the development of a biofilm and the deposit of EPS, pretreatments by sonication and rinsing with SSW were performed on some samples to ensure the access to polymer surface. Experiments performed on the same area before and after sonication showed no change in roughness values (data not shown). Comparison with masking method described above showed that no difference was measured within the experimental uncertainties.

### Contact Angle Measurement

Contact angles (SSW/air/polymer) were measured on each polymer in its initial state (before incubation in seawater) and after 7 days of immersion in SSW, using a profile analysis tensiometer (PAT1M, Sinterface Technologies, Berlin, Germany). We did not measured contact angles after 7 days since surface hydrophobicity was too modified by the conditioning film, as previously observed (Lorite et al., 2011). A series of profiles was acquired for three different droplets of millimetric diameters on each sample during successive advancing and receding stages. All series were analyzed using ImageJ software (version 1.46r, Wayne Rasband, National Institutes of Health, United States) to get the receding and advancing angle in each sample.

### Epifluorescence Microscopy

At each sampling time, one piece of each polymer was rinsed with SSW and fixed for at least 1 h at 4°C with 1% (v/v) glutaraldehyde (final concentration) before freezing. Epifluorescence microscopy observations were done using an Olympus AX-70 PROVIS after 4′,6-diamidino-2-phenylindole (DAPI) staining according to Clays-Josserand et al. (1999). Pictures were taken on 10 fields of each polymer type (Microbe Counter software). The surface areas covered by only bacterial cells and by biofilm (cells + EPS) were determined using the ImageJ software (version 1.46r, Wayne Rasband, National Institutes of Health, United States).

### Flow Cytometry

Three pieces of each polymer were sampled at each sampling time with sterilized forceps and rinsed with SSW. A cell detachment pre-treatment was applied using 1 mmol L^−1^ pyrophosphate during 30 min at room temperature in the dark, followed by a sonication step (3 × 5 s, 40 kHz, 30% amplitude, sterilized probe Branson SLPe). The efficiency of cell-detachment was verified by epifluorescence microscopy before and after cell-detachment, as well as comparison between flow cytometry and epifluorescence microscopy cell counts. The cell detachment pre-treatment was optimized by a set of tests on each polymer substrates. Various mechanical or chemical pre-treatments were tested alone or combined: tetrasodium pyrophosphate (1 and 10 mM); sonication step including a combination of vortex and sonication bath or the use of a sonication probe alone (Branson SLPe, see above); or addition of enzymes mix (Lipase 48 units, beta-galactosidase 10 units, and alpha-glucosidase 0.8 units; Sigma Aldrich). A total of 12 conditions were tested. The chosen protocol was based on a combination of tetrasodium pyrophosphate (1 mM) and sonication probe, which showed the best correspondence between cell counts obtained by flow cytometry and epifluorescence microscopy for the same sample, the latest being 1- to 5-fold higher values than the first. After cell detachment, samples were fixed for at least 1 h at 4°C with 1% (v/v) glutaraldehyde (final concentration) and frozen before further analysis. In parallel, 3×1 mL of seawater (polycarbonate, 47 mm diameter, Whatman) from the control aquarium were also fixed using the same procedure. A 500-μL subsample of the detached cells from plastic or from control seawater was mixed with the nucleic acid dye SYBR Green I (final concentration 0.05% [v/v], Sigma Aldrich) for 15 min, at room temperature and in the dark. Cell counts were performed with a FACSCanto II flow cytometer (BD Bioscience, San Jose, CA, United States) equipped with a blue laser (488-nm, air-cooled, 20-mW solid state), as previously described (Severin et al., 2014).

### Heterotrophic Bacterial Production

Bacterial production (BP) was measured on each polymer type at each sampling time by ^3^H-leucine incorporation into proteins, using a modified protocol from Van Wambeke et al. (2009). Briefly, the same cell detachment pre-treatment protocol as for flow cytometry (see above) was used, based on pyrophosphate together with sonication procedure. This pre-treatment improved the BP signal by a factor from 1.0 to 5.7 compared to control condition with no pre-treatment. This pre-treatment gave also the best results when compared to the other conditions tested (including the combination of vortex and sonication bath and the addition of mix of enzymes, together or alone with the other treatments, see above in the flow cytometry section). Immediately after cell-detachment, ^3^H-leucine (specific activity 112 Ci mmol^−1^; Perkin Elmer) was added at a final concentration of 1 nmol L^−1^ (completed with cold leucine to 150 nmol L^−1^) in triplicate for each sample, which consisted of 1.5 mL of seawater sterilized water containing the piece of plastic together with the detached cells. For seawater samples from the control aquarium, ^3^H-leucine was added at a final concentration of 4.3 nmol L^−1^ to 1.5 mL of control seawater. All samples were incubated in the dark at *in situ* temperature for 3 h. We used the empirical conversion factor of 1.55 ng C pmol^−1^ of incorporated leucine to calculate BP (Simon and Azam, 1989). Cell-specific activities (CSA) were calculated as the ratio between BP and cell counts obtained by flow cytometry.

### DNA Extraction, PCR, and Sequencing

Four replicates of each polymer type were sampled at all sampling times, except for day 15, and stored at -80°C until analysis. In parallel, 1 L seawater was sampled in the control aquarium, successively filtered onto 3 and 0.2 μm pore size polycarbonate filters (47 mm diameter, Nucleopore) and filters were stored at -80°C until analysis. DNA extraction was performed on polymers and filters using a classical phenol-chloroform method for seawater samples (Ghiglione et al., 1999) and a slight modification of the method for polymer samples (Debeljak et al., 2017).

Briefly, the same cell detachment pre-treatment was used as for flow cytometry and BP (see above) before chemical and enzymatic cell lysis (1 mg mL^−1^ lysozyme at 37°C for 45 min followed by 0.2 mg mL^−1^ proteinase K and 1% SDS at 50°C for 1 h). The pre-treatment improved cell lysis since no cells were visible by epifluorescence microscopy after this stage. The molecular size and the purity of the DNA extracts were analyzed using agarose gel electrophoresis (1%) and DNA was quantified by spectrophotometry (GeneQuant II, Pharmacia Biotech).

PCR amplification of the 16S V3–V5 region was done using 515F-Y and 926R primers, particularly well-suited for marine samples according to Parada et al. (2016). Sequencing was performed on Illumina MiSeq by Research and Testing Laboratories (Lubbock, TX, United States). Raw FASTA files were deposited at GenBank under the accession number SRP116996. Sequences were analyzed using Mothur pipeline (Schloss et al., 2009). Paired raw reads were assembled, sequences with homopolymers (>8) and ambiguities were removed and the remaining sequences were aligned using SILVA database. Sequences were trimmed to a same length and a chimera were removed (uchime command). Sequences were classified and operational taxonomic units (OTUs) were defined as clusters sharing 97% sequence identity. Only bacteria were treated in this study, due to the small number of archaeal reads. Chloroplast, mitochondrial and eukaryotic sequences were removed. Bacterial sequences were randomly resampled in the OTU file to enable comparison between samples, by normalizing the number of sequences between samples to the sample with the fewest sequences (*n* = 6,186) using Mothur v.1.38.1. All further analyses were performed on randomly resampled OTU table.

### Statistical Analysis

Alpha-diversity was estimated using the non-parametric, Chao1 species richness estimator from the SPADE software. Simpson, Shannon, and Pielou diversity indexes were obtained using the PRIMER 6 software (PRIMER-E, United Kingdom). Differences between polymers and seawater richness and diversity indexes were tested using a *post-hoc* LSD test after an ANOVA test (Statistica 8.0, Statsoft).

An unweighted-pair group method with arithmetic mean (UPGMA) dendrogram based on Bray–Curtis similarities was used for visualization of beta-diversity. A similarity profile test (SIMPROF, PRIMER 6) was performed on a null hypothesis that a specific sub-cluster can be recreated by permuting the entry species and samples. The significant branch (SIMPROF, *p* < 0.05) was used as a prerequisite for defining bacterial clusters. One-way analysis of similarity (ANOSIM, PRIMER 6) was performed on the same distance matrix to test the null hypothesis that was no difference between bacterial communities of different clusters (Berdjeb et al., 2011). Significant correlations between environmental variables were tracked using Spearman rank pairwise correlations.

## Results

### Polymer Surface Properties

**FIGURE 1:**
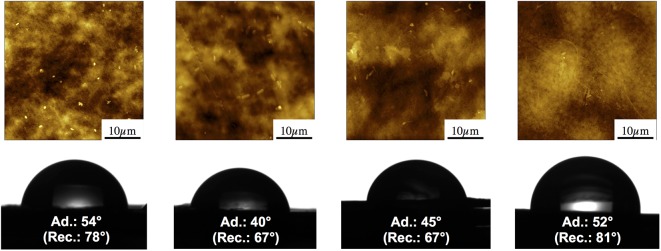
Atomic force microscopy images of PE, OXO, AA-OXO, and PHBV polymer surfaces after a 7-day immersion in seawater, showing different amounts of bacterial cells at the end of the primo-colonization stage (*upper image*). Contact angle drops at advancing point of the four polymers after 7 days of immersion in seawater (receding angle in *brackets*) showing different levels of surface hydrophobicity (*lower image*).

Surface properties of the polymers were derived from AFM data (**Figure 1**). Before incubation in SW, PE, OXO, and AA-OXO presented a rather smooth surface. On the contrary, PHBV showed a rough surface, due to the presence of a spherulitic structure of about 20 μm in diameter. Whereas the first three polymers did not present significant surface modifications with increasing immersion times up to 45 days, the PHBV spherulitic structure went through observable morphological alteration, with clear evidences of swelling and erosion (Supplementary Figure S1).

Root mean square roughness measured on 40 × 40 μm^2^ pictures provided quantitative assessments of surface alterations (**Table 1**). PE, which initially showed the lowest roughness (56 ± 7 nm) at ambient air, did not change significantly for the first 22 days of immersion and slightly increased after 45 days (RMS = 84 ± 9 nm). OXO roughness presented a similar evolution with slightly higher values. AA-OXO roughness remained in the same range as the previous polymers, fluctuating between 63 and 110 nm in the first 22 days. It should be noted that AA-OXO roughness could not be measured at D45 due to a strong bacterial attachment that resisted the washing protocol. PHBV showed the highest initial roughness with 208 ± 21 nm at ambient air. During the incubation period, its value presented large fluctuations over time, with a global increasing trend following important alteration of the initial spherulite structure. The maximum value of RMS was reached after 45 days, where surface erosion (induced most probably by water itself) was clearly visible in AFM micrographs and was then four times higher than that of PE.

**Table 1:** Physical data for the four plastic types (PE, OXO, AA-OXO, and PHBV) according to immersion time in days (D), including roughness (RMS, in nm), contact angle (CA, receding – advancing, in degree) and carbonyl index (CI).

		DO	D7	D15	D22	D30	D45
PE	RMS	56	49		46		84
	CA	85–94	54–78				
	CI		0.74	0.48	0.39	0.56	0.57
OXO	RMS	87	122		106		112
	CA	61–79	40–67				
	CI		0.49	0.5	0.39	0.47	0.85
AA-OXO	RMS	110	63		64		ND
	CA	52-76	45–67				
PHBV	RMS	208	129	322	240		358
	CA	78–99	52–81				

Advancing and receding contact angles (SW/air/polymer) were measured on initial dry samples and after 7 days of immersion in SW (**Figure 1** and **Table 1**). Initially, all polymers presented a rather hydrophobic surface with receding and advancing contact angle close to 90°, with PHBV and PE being the most hydrophobic. The addition of polar groups from PE to OXO and AA-OXO explains their lower hydrophobicity. The contact angle hysteresis (difference between receding and advancing contact angle), which is directly related to the roughness or the chemical heterogeneity of a surface, showed higher values for OXO and AA-OXO compared to PE, in agreement with the more homogeneous chemical composition of the latter. PHBV showed a large hysteresis, probably reflecting its structuration in big spherulites, in agreement with AFM observation and roughness measurements (Supplementary Figure S1). After immersion, the decrease in hydrophobicity for all polymers can be connected to surface reconstruction for OXO and AA-OXO, surface reconstruction and water swelling for PHBV and probably adsorption of polar molecules on the surface in the case of PE.

### Dynamics of Bacterial Cell Counts on Polymers and in Seawater

Epifluorescence microscopy observations were not possible for AA-OXO and PHBV samples, because of strong auto-fluorescence background under UV light for these two polymers. Because our cell detachment pre-treatment showed that flow cytometry approach was possible for all polymer and it slightly underestimated cell counts as compared to epifluorescence microscopy by a factor of 1 to 5, we decided to use the flow cytometry cell counts to provide comparable data obtained with the same technique. Then, epifluorescence microscopy was used only to confirm the results obtained by flow cytometry and to estimate plastic surface area covered by bacterial cells, when available (only for PE and OXO).

Flow cytometry data highlighted three distinct phases of biofilm formation for all polymer types: primo-colonization, growth, and maturation (**Table 2**). Primo-colonization lasted for the first 7 days following immersion, with cell counts being, respectively, 1.5, 1.6, and 1.3 × 10^5^ cells cm^−2^ for PE, OXO, and PHBV and 9.3×10^5^ cells cm^−2^ for AA-OXO. Cell counts increased on all polymers during the growing phase, but at different rates: after 15 and 22 days, cell counts on PHBV and AA-OXO biofilms were about fivefold more than that on PE and OXO. The stabilization phase was visible after 22 days for PE, OXO, and PHBV, reaching, respectively, 3.7, 6.9, and 16.3 × 10^5^ cells cm^−2^ at the end of the experiment, whereas cell counts continued to increase for AA-OXO to finally reach 34.1 × 10^5^ cells cm^−2^.

**Table 2:** Biological data for the four plastic types (PE, OXO, AA-OXO, and PHBV) compared to seawater (SW) according to immersion time in days (D), including bacterial cell count (BC, ×10^5^ cell mL^−1^ for SW or ×10^5^ cell cm^−2^ for plastic samples), bacterial production (BP, in ngC L^−1^ h^−1^ for SW or ngC dm^−2^ h^−1^ for plastic samples), and bacterial specific activity (SA, ×10^−3^ fgC cell^−1^ h^−1^).

		D7	D15	D22	D30	D45
SW	BC	1.16	0.89	1.71	1.15	3.07
		*(0.03)*	*(0.04)*	*(0.11)*	*(0.001)*	*(0.14)*
	BP	9.09	10.5	16.8	21.3	41
		*(0.8)*	*(1.1)*	*(0.7)*	*(2.0)*	*(1.8)*
	SA	0.079	0.118	0.098	0.185	0.133
PE	BC	1.53	3.4	6.76	9.05	3.7
		*(0.34)*	*(0.51)*	*(1.45)*	*(1.23)*	*(1.33)*
	BP	38.5	352.3	426.7	29	55.6
		*(20.4)*	*(114.9)*	*(241.5)*	*(13.5)*	*(36.7)*
	SA	2.52	10.37	6.33	0.32	1.50
	cov	1.00%	5.1%	6.5 %	15.1%	29.2 %
OXO	BC	1.57	3.75	5.65	4.92	6.89
		*(0.34)*	*(1.29)*	*(1.27)*	*(1.35)*	*(1.40)*
	BP	105.5	178.7	217.2	60.3	55.3
		*(36.2)*	*(4.0)*	*(10.2)*	*(21.4)*	*(12.2)*
	SA	6.74	5.02	3.85	1.23	0.80
	cov	3.4%	3.4	10.1%	12.3%	18.1%
AA-OXO	BC	9.25	16.1	28.4		34.1
		*(3.50)*	*(4.47)*	*(3.86)*		*(8.47)*
	BP	145.9	1396.9	1369.7		131.4
		*(20.1)*	*(90.0)*	*(193.6)*		*(7.25)*
	SA	1.58	8.67	4.82		0.39
PHBV	BC	1.25	15.2	15.3		16.3
		*(0.37)*	*(2.92)*	*(3.94)*		*(3.61)*
	BP	67	1090.4	259.4		240.9
		*(58.4)*	*(513.5)*	*(125.9)*		*(100.4)*
	SA	5.38	7.19	1.70		1.47

**FIGURE 2:**
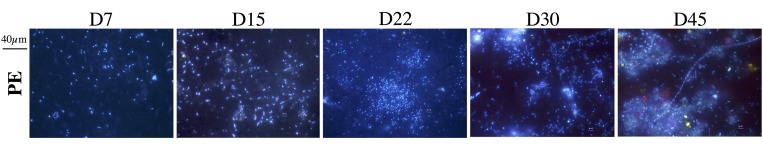
Epifluorescence micrographs of DAPI-stained PE plastics after 7, 15, 22, 30, and 45 days of immersion in seawater. Bar: 40 μm.

The three phases were also visually observed by epifluorescence microscopy (**Figure 2**). Primo-colonization was characterized by single cells spreading out homogenously on the surface resulting in cell coverage of 1 and 3% of the PE and OXO surface at day 7, respectively (**Table 2**). Cell abundance increased unevenly during the growing phase, leading to a patchy distribution of cell aggregates on both PE and OXO films, representing, respectively, 6.5 and 10.1% coverage at day 22. Together with an increase in exuded EPS clearly visible on micrographs after day 22, the biofilm coverage on the surface reached 29.2 % and 18.1% after 45 days for PE and OXO, respectively (**Table 2**).

### Dynamics of Bacterial Community Structure and Diversity on Polymers and in Seawater

**FIGURE 3:**
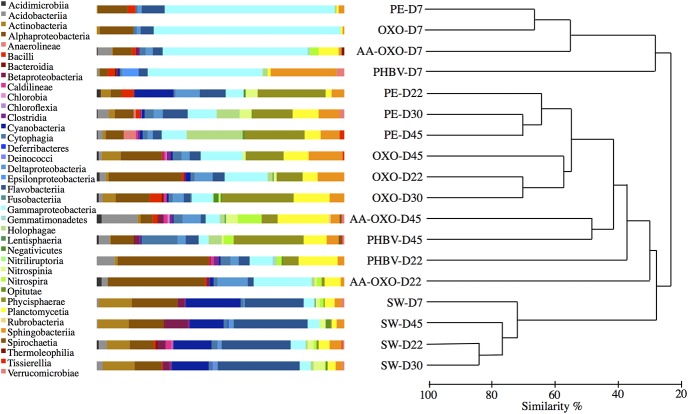
Comparison of taxonomic abundances and community structure of bacteria in seawater (SW) and attached in the four plastics (PE, OXO, AA-OXO, and PHBV) according to immersion time in days (D), by cumulative bar charts comparing relative class abundances (*left*) and by UPGMA dendrogram based on Bray–Curtis similarities between sequencing profiles (*right*).

Next-generation DNA sequencing resulted in 265,998 tags falling into 823 bacterial OTUs at 97% similarity level, after randomly resampling to 6,186 sequences per sample to provide statistical robustness when comparing diversity between samples. The cluster analysis showed a clear dissimilarity (>70%) between seawater controls and polymer samples during the course of the experiment (**Figure 3**). Overall, bacterial community structure on all polymer types showed spectacular changes, first in the diversity of bacteria that colonized the polymers compared with the surrounding seawater, and second in the growing and maturation phases compared to the primo-colonization phase. All polymer types sampled at day 7 clustered together in a group showing low similarity (<25%) with other samples (**Figure 3**). Within this cluster, the PHBV community structure significantly differed (SIMPROF test) from the other polymer types. The temporal dynamics of the bacterial assemblages during the growing and maturation phases differed with the polymer type. PE and OXO biofilms formed distinct, yet close sub-clusters and showed few changes from days 22 to 45. Conversely, both communities from AA-OXO and PHBV presented strong changes during this period (<40% similarity from days 22 to 45 between samples from the same polymer type).

Overall, the observed changes in the diversity indexes (Shannon, Pielou, Chao1, and Simpson; Supplementary Table S1) were related to the polymer type (ANOVA test, *p* < 0.05), but not to incubation time: we could not find any relation between the changes in diversity indexes and the different stages of biofilm formation. The equitability (Pielou) on PE was significantly higher than on AA-OXO, PHBV and seawater (LSD test, *p*-value < 0.05) (Supplementary Table S1). The Shannon diversity index was also higher on PE compared to AA-OXO (LSD test, *p*-value < 0.05). The Chao1 index ranged from 113 (OXO at day 7) to 322 (PHBV at day 45).

**FIGURE 4:**
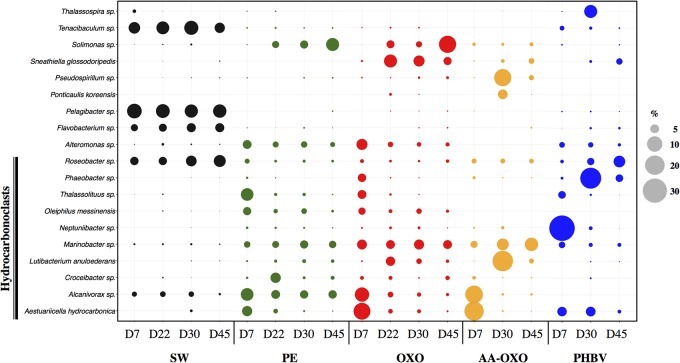
Bubble plot showing the relative abundance (%) of the majority OTUs (>5%) in each compared sample between immersion times in days (D) in seawater (SW) and in the four plastic types (PE, OXO, AA-OXO, and PHBV). Putative hydrocarbonoclastic OTUs were *highlighted*.

Taxonomic analyses confirmed the specificity of the community structures formed on the polymers compared to seawater, the latter being dominated by Alphaproteobacteria, Flavobacteria, Cyanobacteria, and Actinobacteria throughout the experimentation (**Figure 3**). On all four polymers type, the primo-colonizers belonged to Gammaproteobacteria, which represented between 45 and 75% of the total OTU in each community (**Figure 3**). On PE, OXO and AA-OXO, this group was mainly dominated by *Alcanivorax* sp., *Aestuariicella hydrocarbonica, Alteromonas* sp., and *Thalassolituus* sp. followed by *Marinobacter* sp. and *Maricurvus* (**Figure 4**). On PHBV, *Neptiniibacter* sp. made up for more than 30% of the community, while this OTU remained undetected on all other polymers.

The growing and maturation phases were characterized by few changes on PE and OXO samples, where *Croceibacter* sp. was the dominant OTU on PE, whereas *Sneathiella glossodoripedis* dominated on OXO (**Figure 4**). Only a significant increase of *Solimonas* sp. occurred during the stabilization phase on OXO. More changes were observed on AA-OXO and PHBV during the growing and maturation phases, with large dissimilarities between sampling time. The OTUs *Lutibacterium anuloederans* and *Pseudospirillum* sp. were found in high amounts on AA-OXO during the growth stage, whereas *Phaeobacter* sp. stand out on PHBV. The majority of OTUs identified at day 22 on AA-OXO and PHBV decreased at day 45, giving way to a higher abundance of unclassified OTUs. During the maturation phase, Gammaproteobacteria decreased and Alphaproteobacteria increased proportionally, with Phycisphaerae, Planctomycetia, and Sphingobacteriia classes in particular whatever the plastic type (**Figure 3**).

### Presence of Putative Hydrocarbonoclastic Bacteria

We identified 34.4% of the total sequences on polymer samples as being putative hydrocarbonoclastic bacteria (HCB), compared to 4.1% in control seawater. Among the most abundant OTUs per polymer sample (>5% of the total OTUs in one sample), we found the HCB *Alcanivorax* sp., *Aestuariicella hydrocarbonica, Marinobacter* sp., *Lutibacterium anuloederans*, and *Neptuniibacter* sp. (**Figure 4**). SIMPER analysis showed that these 5 OTUs explained more than 13% of the dissimilarity between polymers and seawater communities. Overall, HCB were particularly abundant in bacterial communities during the primo-colonization phase on all polymer types (1.7 to 3-fold more HCB were identified on polymers compared to seawater) and generally decreased afterward. *Aestuariicella hydrocarbonica* was found in higher abundance on all polymer types, reaching up to 20 and 24% of sequences in OXO and AA-OXO, respectively. *Alcanivorax* sp. reached similar relative abundances, but was not detected on PHBV, where HCB were instead dominated by *Neptuniibacter.* These three OTUs decreased after day 7 and were replaced by another HCB, such as *Marinobacter* on PE, OXO and AA-OXO, and *Lutibacterium anuloederans* on AA-OXO.

### Presence of Putative Pathogenic Bacteria

We identified 23 putative pathogen OTUs in all our samples, which represented <3% (3,817 sequences) of the total sequences (plastic and seawater samples). A 80% of putative pathogen OTUs were found in seawater samples (mainly *Tenacibaculum* sp.). On plastic samples, half of the putative pathogen OTUs belonged to *Vibrio* sp., 20% being identified as *Tenacibaculum* sp. and 11% as *Staphylococcus aureus.* Overall, the abundance of putative pathogenic OTUs remained steady during the different biofilm stages, except for PHBV showing two times more putative pathogen OTUs during primo-colonization.

### Heterotrophic BP and CSA on Polymers and in Seawater

During the primo-colonization stage, BP were in the same order of magnitude between the four polymers (from 38.5 ngC dm^−2^ h^−1^ on PE to 145.9 ngC dm^−2^ h^−1^ on AA-OXO) (**Table 1**). The temporal dynamics of BP on PE and OXO were comparable, peaking during the growing phase (426.7 and 217.2 ngC dm^−2^ h^−1^ at day 22, respectively) and decreasing during the maturation phase (55.6 to 55.3 ngC dm^−2^ h^−1^ at day 45, respectively). AA-OXO and PHBV biofilms presented different trends. BP peaked at day 15 for both AA-OXO and PHBV (1396.9 and 1090.4 ngC dm^−2^ h^−1^, respectively), being until eightfold higher than PE and OXO. PHBV biofilm became less active at day 22, reaching a plateau around 250 ngC dm^−2^ h^−1^ until day 45. AA-OXO kept a high activity until day 22 (1396.7 ngC dm^−2^ h^−1^) and decreased drastically at day 45 (131.4 ngC dm^−2^ h^−1^). Seawater BP remained lower than polymers BP throughout the experiment rising from 9.09 ngC L^−1^ h^−1^ at D7 to 41 ngC L^−1^ h^−1^ at D45.

Cell-specific activity was very high on plastic compared to free-living bacteria (maximum of 10.37 and 0.13 × 10^−3^ fgC cell^−1^ h^−1^, respectively) and especially during the growing phase of the biofilm on plastics (from 43- to 88-fold higher than in seawater). Indeed, cell-specific activity peaked at day 15 but decreased generally after 22 days on plastic, whereas it changed more randomly in seawater.

## Discussion

In the present study, we show that plastic polymers with different composition, when immersed under identical marine conditions, are first colonized by similar bacterial communities to constitute support matrices for the formation of contrasted biofilms with dissimilar diversities and activities, growth efficiency, and maturation properties. We also investigated the possible relation between surface properties and bacterial cell counts on plastics, speculated to be a key factor controlling biofilm formation (Pasmore et al., 2002).

### Succession of Biofilm Colonization Phases on Polymers

In this study, we observed three typical successive phases of biofilm formation on artificial surfaces: initial, growth and maturation phases. The initial phase lasted for the first week of immersion and was characterized by an abundant and homogeneous bacterial colonization on all polymers within the first 7 days of incubation, with a cell density ranging from 1.25 × 10^5^ to 9.25×10^5^ cell cm^−2^. The growing phase (after day 7 to 22) significantly differed between non-biodegradable (PE and OXO) and biodegradable (AA-OXO and PHBV) polymers, with a higher biomass increase on the latter. At this stage, cells formed aggregates and biofilms became more patchy, as also observed on plastic marine debris in the North Pacific Gyre (Webb et al., 2009; Carson et al., 2013) and in the Mediterranean Sea (Dussud et al., 2018). The stabilization phase generally occurred after 3 weeks (from day 22 to 45) with the highest cell abundance reached on AA-OXO and PHBV, being more than five times higher than that accumulated on the non-biodegradable PE and OXO. These results are in accordance with Lobelle and Cunliffe (2011) reporting stabilization phase within a month on PE-based food bags, even if their results were based on cultivable bacteria that greatly underestimate cell counts of the entire biofilm (Ferguson et al., 1984). Other studies evaluated cell abundance using SEM, AFM, or epifluorescence microscopy (Harrison et al., 2014; Bryant et al., 2016), but none of them provided direct cell counts. As far as we know, this study presents the first results of direct cell counts on polymers using flow cytometry coupled with epifluorescence microscopy. It should be emphasized that epifluorescence microcopy was not usable for some polymers due to strong auto-fluorescence background (i.e., AA-OXO and PHBV), whereas our cell detachment pre-treatment permits to use flow cytometry as accurate technique to estimate cell counts in all polymers. When possible, the comparison of the two techniques showed systematic underestimation of cell counts for flow cytometry by a factor of 1 to 5, which is consistent with previous studies on organic particle-attached bacteria (Worm et al., 2001; Mével et al., 2008).

We also explored the possible relation between polymer surface characteristics and microbial colonization. This is a complex question, since several effects need to be considered at once: the chemical nature (Lorite et al., 2011; Siddiqa et al., 2015), roughness (Riedewald, 2006), and heterogeneity (Morra and Cassinelli, 1997) of the polymer surface on the one hand, and the potential hindrance of these properties by the microbial conditioning film (Lorite et al., 2011), on the other hand. Moreover, polymers are known to alter their properties when immersed in water, due to water diffusion or reconstruction of their surface in order to minimize the interfacial energy. Indeed, we observed here a decrease in hydrophobicity for all polymers after 7 days of immersion in seawater. This complexity might explain why there is still no consensus today, as to whether, for instance, a hydrophobic surface will increase or not bacterial adhesion (Morra and Cassinelli, 1997). Several articles on biofouling nevertheless acknowledge that high-energy surfaces (“hydrophilic surfaces”) tend to favor biofilm growth (Callow and Fletcher, 1994; Artham et al., 2009). Our study presents the first results combining the observation of successive biofilm colonization phases on plastics together with the evolution of their surface roughness, contact angles and hysteresis before and after immersion in seawater. When comparing the three types of PE-based polymers, we clearly observed that colonization increased with increasing polarity (AA-OXO > OXO > PE) for similar roughness. In the same way, colonization was higher for PHBV than for PE, probably because PHBV is more polar, even though its roughness was larger than that of PE. A clear conclusion that can be drawn from these results is that the surface polarity has definitely an impact on colonization at sea, whether through the adsorption of a more abundant or different conditioning film, or directly through attracting more bacteria. Finally, one should keep in mind that cells numbers reflect not only their rate of adhesion but also the multiplication/disappearance rate of the different species, which can be affected in the case of biodegradable substrates where plastic is not only a physical support matrix but also a potential source of nutrients for bacteria. A hint into these rates is given by the measured activity and diversity of the bacterial colonies which are discussed thereafter.

### Bacterial Community Succession on Polymers

The bacterial communities accumulated on the polymer surfaces differed from those in the seawater during the entire course of the experiment. This assessment is in line with previous studies revealing a clear niche partitioning between bacteria living on plastics versus surrounding seawaters (Zettler et al., 2013; Amaral-Zettler et al., 2015; Dussud et al., 2018). Our experimental conditions did not disrupt the natural assemblages of seawater bacteria circulating in the aquarium during the course of the experiment, as observed in the control aquarium that did not contain plastic. Together with the slight changes observed in bacterial abundance in the control aquarium which are in line with values commonly found in the Mediterranean Sea (Pulido-Villena et al., 2011), these results validated our capability to maintain natural conditions for 45 days in an experimental setup renewed with natural seawater every 30 min.

Primo-colonizers of the plastics represented <0.1% of the bacterial diversity found in the water, corresponding to the less abundant or rare taxa that make up a substantial portion of bacterial communities in the oceans and constitute the so called “rare biosphere” (Sogin et al., 2006). These results demonstrate that the “seed bank” theory (Pedrós-Alió, 2012; Sauret et al., 2014) applies particularly well to the early colonizers and to the plastisphere in general. Members of the bacterial communities living on plastics, although rare in the seawater, prove here to be opportunistic species able to grow and to become the “core species” living on plastics. Overall, we found that Gammaproteobacteria dominated primo-colonizers on all polymer types, as already reported for the early colonization of PE (Harrison et al., 2014; De Tender et al., 2017). This taxonomic group was also identified as a family of primo-colonizers on other artificial surfaces in coastal waters such as acryl, glass, steel, or filtration membranes from drinking water treatment plants (Hörsch et al., 2005; Lee et al., 2008). The bacterial community structures of primo-colonizers were similar between all polymer types, except for PHBV, for which bacteria belonged to the same cluster but presented much less similarity and were largely dominated by *Neptuniibacter* sp.

In the next phase of biofilm growth and during the maturation phase, we observed a clear distinction between bacterial communities growing on non-biodegradable and biodegradable polymers. While PE and OXO eventually hosted a homogeneous cluster, the community structures on AA-OXO and PHBV continued to change over time. Previous studies also underlined rapid shifts in bacterial communities between the initial and successive colonization phases on other artificial surfaces, such as polyurethane painted plastics (Dang and Lovell, 2000), desalination plant system (Elifantz et al., 2013) or on acryl, glass and still coupons (Lee et al., 2008). With time, we observed that members of the class Alphaproteobacteria became increasingly abundant whatever the polymer type and remained distinct from the communities living in the control seawater.

Our study compared for the first time the dynamics of marine bacterial communities on polymers of similar chemical basic formulation (i.e., PE-based) but with d2w additives (Symphony Environmental Technology) with or without pre-aging. The cluster analysis showed that similar communities dominated the non-biodegradable PE and OXO during the growing and maturation phases, but differed drastically from the biodegradable AA-OXO. Difference in bacterial community structure may be explained by surface properties, since AA-OXO present higher oxidation state, lower hydrophobicity compared to PE and OXO.

The two biodegradable polymers AA-OXO and PHBV continued to change over the growing and maturation phases of the biofilm. Polymer degradation is considered to proceed through several stages (i.e., biodeterioration, biofragmentation, assimilation, and mineralization), which result from complex synergetic interactions between bacterial communities that also change over the biodegradation process (Lucas et al., 2008; Dussud and Ghiglione, 2014). Even if biodegradation processes occurring in both AA-OXO and PHBV are becoming better understood for bacteria cultured in the laboratory (Deroiné et al., 2015; Eyheraguibel et al., 2017), further studies are needed to describe the complex interactions between bacterial communities in the biofilm and their role in plastic biodegradation in natural conditions.

### Potential Bacterial Degradation of Complex Carbon Molecules in Plastics

The SIMPER analysis revealed a clear dominance of putative HCB on plastic compared to seawater. Their presence on the plastic surface has been observed in various neustonic debris (mainly of PE and PP composition) in the North Pacific Gyre (Zettler et al., 2013; Debroas et al., 2017) and in the Mediterranean Sea (Dussud et al., 2018), or on 5- to 6-weeks immersed PET drinking water bottles (Oberbeckmann et al., 2016). All these authors postulated that these plastic-dwelling microbes possessed the metabolic potential to degrade plastics and/or plastic-bound organic pollutants. Such hypothesis was recently supported by metagenomic analyses highlighting an overexpression of xenobiotic degradation functions by plastisphere communities in the North Pacific Gyre (Bryant et al., 2016).

Another hypothesis is the capability of HCB to overcome the poor accessibility of hydrophobic substrates, which may play a crucial role in the early colonization phase on hydrocarbon-based plastics (Lobelle and Cunliffe, 2011). Biofilm formation at the hydrocarbon–water interface has been observed with various alkane-degrading strains including *Oleiphilus messinensis* (Golyshin et al., 2002) and *Marinobacter* sp. (Vaysse et al., 2009), which dominated the early colonization phase on PE, PE-OXO, and AA-OXO in our study, together with other known alkane-degraders *Alcanivorax* sp. (Yakimov et al., 2007) and *Aestuariicella hydrocarbonica* (Lo et al., 2015). Biofilm formation has been shown to promote growth at the hydrocarbon–water interface by facilitating interfacial access, thus constituting an efficient adaptive strategy for assimilating hydrocarbon (Bouchez-Naïtali et al., 2001).

If putative HCB dominated on hydrocarbon-based plastics (PE, OXO, and AA-OXO), PHBV showed instead a succession of PHA-degraders. Indeed, members of *Neptuniibacter* sp. (Chen et al., 2012), *Phaeobacter* sp. (Frank et al., 2014), and *Roseobacter* sp. (Xiao and Jiao, 2011) previously shown to present the capability to accumulate or metabolize PHA, were dominant in the early colonization, growth and maturation phases, respectively. Further biodegradation studies in natural environment are needed to further describe the role of these species in PHA polymers degradation.

### A High and Variable Heterotrophic BP on Polymers

Our study provides the first BP data on polymers. A high temporal variability of BP was found during the successive phases of biofilm formation on each polymer. Overall, BP peaked after 2 weeks during the growing phase in all polymer types (from day 15 to 22), where CSA were the highest, and both parameters decreasing in the maturation phase.

In our seawater circulation system, BP reached 41 ngC L^−1^ h^−1^, a value similar to what is generally reported *in situ* in the NW Mediterranean Sea (Lemée et al., 2002), thus making extrapolation of our results to natural seawater possible.

Comparing BA and BP data between polymer films (in cm^−2^) and seawater (in mL^−1^) was irrelevant because one is counted in a volume and the other one on a surface, but using cell-specific activity (in ngC cell^−1^ h^−1^ for both plastic and seawater) made this comparison possible. We then found that bacteria attached on polymer were particularly active compared to the free-living bacteria, the cell-specific activity being from 43- to 88-fold higher especially during the growing phase in the polymers. Such difference may be explained by the presence of labile inorganic and organic matter on the plastic, as on any solid surface immerged in seawater (Cooksey and Wigglesworth-Cooksey, 1995). Another explanation could be that biodegradation has started on some polymers, since they can theoretically be used as carbon source by bacteria (Dussud and Ghiglione, 2014). The BP observed on the two biodegradable polymers (AA-OXO and PHBV) proved until 30 times higher than that measured on non-biodegradable polymers support this hypothesis. Unfortunately, no specific biodegradation assays on organic matter or plastic were performed in this study, which may help to test these hypotheses and their complementarity. Further studies are needed to differentiate organic matter utilization from polymer biodegradation when measuring BP on plastics.

In this paper, we did not evaluate the biodegradability of the polymers tested during our experiment. Nethertheless, a better understanding of the biofilm forming on plastic in natural conditions is necessary to develop realistic tests of biodegradation. A very recent review pointed that current standards and test methods are insufficient in their ability to realistically predict the biodegradability of plastics in aquatic environment (Harrison et al., 2018). In particular, the type of inoculum and the presence of organic matter are potential sources of uncertainties on the biodegradability tests, generally based on respirometric measurements (Sharabi and Bartha, 1993). For example, a study on PHBV aged film demonstrated a large loss of weight after 180 days in natural seawater and a biodegradation by respirometry (Deroiné et al., 2015). To complete this study a characterization of the microorganisms diversity would have been important to better understand the mechanisms of PHBV biodegradation in seawater. Differences in the oxidation degree of the polymers, in the environmental conditions or in the methodologies used are also important factors that may explain controversary results showing ever no significant proof of mineralization of pre-oxidized OXO in marine water (Alvarez-Zeferino et al., 2015) or clear biodegradation in other environments (Jakubowicz, 2003; Chiellini et al., 2007; Ojeda et al., 2009; Yashchuk et al., 2012; Eyheraguibel et al., 2018). Giving the fact that relatively few studies focused on colonization of plastic at sea, this study should help further researches on biodegradability of plastics in marine habitats.

## Author Contributions

CD and CH have conceived and designed the study. CD, CH, MG, and PF acquired the data. CD, CH, MG, PF, and J-FG analyzed and interpreted the data. J-FG, PH, SB, PF, and MG provided the equipment. CD and J-FG drafted the manuscript. PF, MG, and A-MD critically revised the manuscript for important intellectual content. BE, A-LM, JJ, JC, NC, CO, and SR approved the version of the manuscript to be published.

## Conflict of Interest Statement

The authors declare that the research was conducted in the absence of any commercial or financial relationships that could be construed as a potential conflict of interest.
