# Supplementary figures and images for: Colonization of Non-biodegradable and Biodegradable Plastics by Marine Microorganisms
Source: Front Microbiol. 2018 Jul 18;9:1571. doi: 10.3389/fmicb.2018.01571 (PMC6058052; doi:10.3389/fmicb.2018.01571)

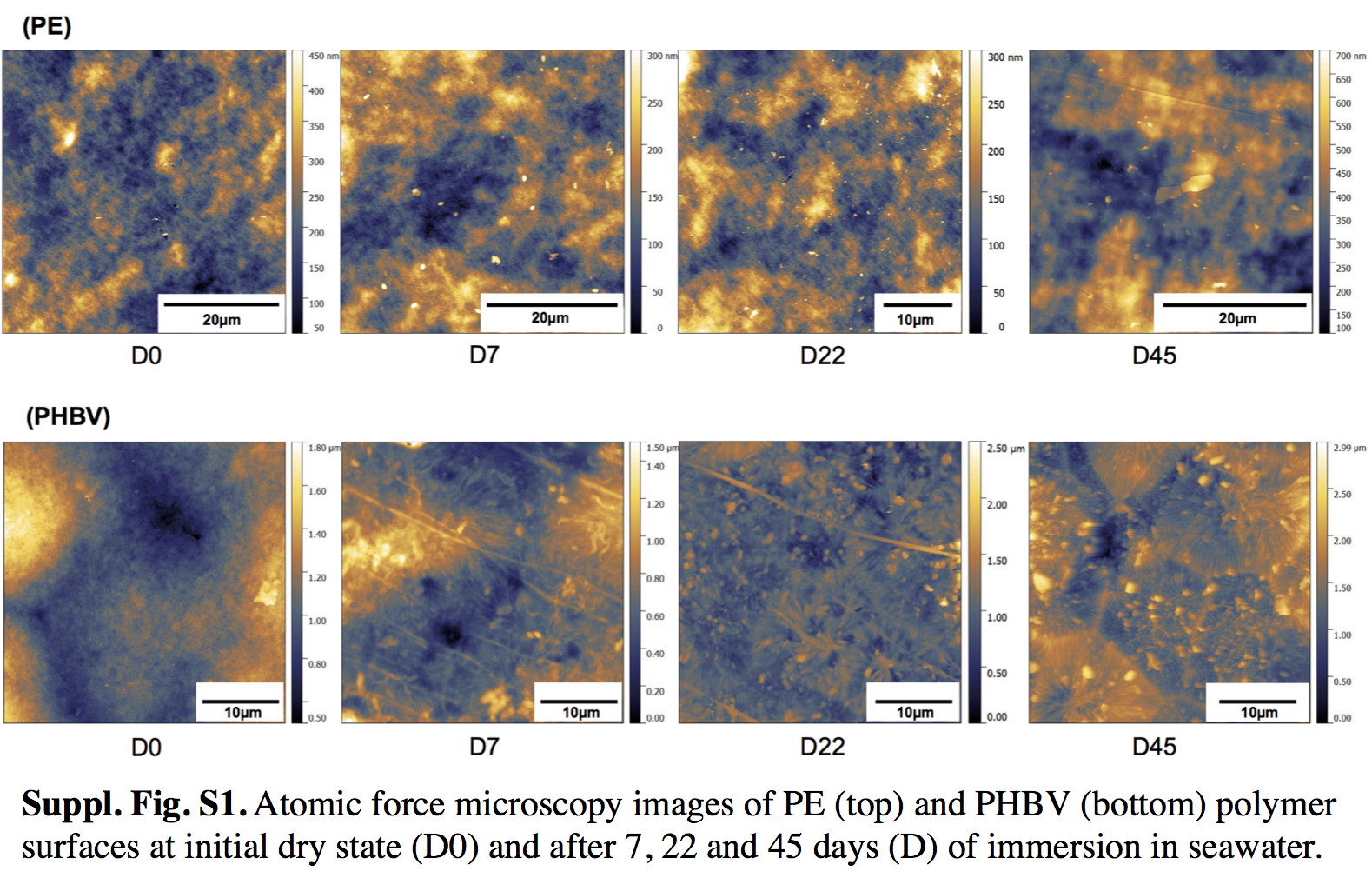

Supplement: Supplementary file 1 [file Image_1.TIFF]
