# Supplementary material for: Colonization of Non-biodegradable and Biodegradable Plastics by Marine Microorganisms
Source: Front Microbiol. 2018 Jul 18;9:1571. doi: 10.3389/fmicb.2018.01571 (PMC6058052; doi:10.3389/fmicb.2018.01571)
Supplement: Supplementary file 2 [file Table_1.pdf]

Suppl. Table S1: Number of reads for tag sequencing and alpha-diversity indexes (Chao1, Pielou, Shannon, Simpson) for the 4 plastic types (PE, OXO, AA-OXO, PHBV) and seawater (SW) samples. Underlined values correspond to the minimum; bold values correspond to the maximum.

Sample	Day	Number of reads (N)	Chao 1	Pielou	Shannon	Simpson (1-λ)
SW	D7	178	259,4	0,616	3,191	0,920
	D22	195	292,5	0,594	3,129	0,910
	D30	171	230,4	0,605	3,111	0,916
	D45	150	201,0	0,607	3,039	0,917
PE	D7	146	185,0	0,660	3,288	0,929
	D22	111	124,0	**0,770**	3,628	0,953
	D30	202	229,3	0,734	**3,894**	**0,958**
	D45	154	183,5	0,710	3,577	0,938
OXO	D7	97	112,8	0,658	3,009	0,914
	D22	175	211,4	0,672	3,473	0,937
	D30	199	250,0	0,668	3,535	0,928
	D45	171	217,4	0,638	3,283	0,920
AA-OXO	D7	183	249,5	0,572	2,98	0,881
	D22	158	222,2	0,550	2,784	0,867
	D45	199	228,3	0,642	3,396	0,916
PHBV	D7	139	152,0	0,608	3,002	0,873
	D22	**214**	271,6	0,642	3,447	0,926
	D45	208	**322,0**	0,601	3,206	0,863
